# Can mirror reading reverse the flow of time? Evidence from Japanese speakers

**DOI:** 10.1186/s41155-020-00156-7

**Published:** 2020-08-11

**Authors:** Wenxing Yang, Xueqin Feng, Jing’ai Jin, Yuting Liu, Ying Sun

**Affiliations:** College of Foreign Studies, Yangzhou University, 196 West Huayang Road, Yangzhou, 225127 Jiangsu Province China

**Keywords:** Space, Time, Writing direction, Representational flexibility, Japanese

## Abstract

Accumulating evidence over the last two decades has established the causal role of a unidirectional orthography in shaping speakers’ mental representations of time. Casasanto and Bottini (Journal of Experimental Psychology: General, 143, 473-479, 2014) extended previous findings by showing that exposure to mirror-reversed orthography of speakers’ native language could completely redirect their mental timelines within minutes. However, the question of whether such a causal effect of writing direction on temporal cognition can be identified in speakers whose native languages adopt bidirectional orthographies remains underexplored in the literature. To address this issue, the present study focused on Japanese which uses bidirectional writing systems, one proceeding horizontally from left to right (HLR) and one vertically from top to bottom (VTB). Two experiments were performed, and the tasks asked participants to process standard/mirror orthography prime questions about time arranged horizontally or vertically, followed by horizontal or vertical arrays of pictorial target stimuli about temporal relations. Results demonstrated that Japanese speakers encoded passage of time into a top-to-bottom linear path commensurate with the VTB writing direction, but they did not align their mental representations of time with the HLR writing orientation. Accordingly, exposure to mirror-reversed bidirectional orthographies redirected Japanese speakers’ vertical but not horizontal space-time mappings. Theoretical implications concerning the causal effects of bidirectional orthographies and the generalizability of the representational flexibility of time maintained by Casasanto and Bottini (Journal of Experimental Psychology: General, 143, 473-479) are discussed.

## Introduction

Time and space can hardly be teased apart in language and the human mind. Across many languages, spatiotemporal metaphors for temporal sequences indicate that passage of time unfolds along a sagittal (front-back) axis: the future is *ahead* and the past is *behind* us. Inspired by patterns in metaphorical language (Lakoff & Johnson, 1980, 1999), people have been documented to rely on front/back spatial representations to think about time in several psychological experiments (Boroditsky, 2000; Miles et al., 2010; Torralbo et al., 2006; Ulrich et al., 2012; Walker et al., 2017). Nonetheless, space and time are linked in the mind in more ways than linguistic analyses alone can reveal. The way people use space to talk about time is not necessarily the way they use space to think about it (Casasanto, 2016). The sagittal axis is not the only mental timeline that people possess. In fact, it may not even be the dominant spatial axis through which speakers of different languages represent time.

To date, the horizontal (left-right) axis is not conventionalized in any known spoken languages. Despite the total absence of left-right space-time mappings in languages, there is robust evidence that people across cultures display salient propensities to represent time along the horizontal (left-right) axis and that the orientation of the mental timelines correlates with the direction in which people read and write. For instance, speakers of many Indo-European languages (e.g., English, Spanish, Dutch, Italian) which are written horizontally from left to right (HLR) created spatial representations of time in line with the writing direction of their respective native languages, i.e., conceptualizing earlier/past event or time-point as leftward and later/future event or time-point as rightward (Bergen & Chan Lau, 2012; Casasanto & Bottini, 2014; Fuhrman et al., 2011; Ouellet et al., 2010; Santiago et al., 2010; Vallesi et al., 2014; Weger & Pratt, 2008; Yang & Sun, 2016a). On the contrary, speakers of other languages (e.g., Arabic, Hebrew) written horizontally from right to left (HRL) exhibited an opposite tendency as compared with speakers of the abovementioned Indo-European languages, given that they encoded time flow into a right-to-left spatial coordinate (Fuhrman & Boroditsky, 2010; Tversky et al., 1991). Therefore, studies investigating the relationship between directionalities of people’s space-time associations and writing orientations appeared to produce consistent results, because orthographies were confirmed to determine whether people access left-to-right or right-to-left mental timelines.

Among the foregoing empirical research, one influential work which received 118 citations in Google Scholar is Casasanto and Bottini (2014). This study not only showed that reading and writing direction can enact a causal role in shaping people’s implicit time representations, but also extended previous investigations by demonstrating that changing the orientation of the standard orthography in speakers’ native language could completely redirect people’s mental timelines. The tasks in Casasanto and Bottini (2014) were designed as follows. Native speakers of Dutch saw past-oriented phrases like “een jaar daarvoor” (a year before) and future-oriented phrases like “een dag daarna” (a day after) on the computer screen. Participants had to judge if the phrase stood for the past or future time point by pressing one of two keys on a keyboard. The canonical block designated the left key as “past” and the right key as “future” while the non-canonical block reversed the key mappings. Half of the participants were assigned to the standard orthography condition in which all instructions and stimuli appeared in the ordinary HLR Dutch orthography. The other half of the participants were assigned to the mirror-orthography condition in which instructions and stimuli were presented in mirror-reversed (HRL) Dutch. Participants in the standard orthography condition were faster to make a decision when the locations of the response keys matched a left/past-to-right/future timeline as compared with the opposite key mapping, whereas this effect was reversed for participants in the mirror-reversed condition, i.e., reaction times (RTs) were faster with the right/past-left/future than with the left/past-right/future key arrangement. This was counted as evidence indicating the causal role of writing/reading direction in structuring people’s mental timelines. Specifically, this study suggested that a few minutes’ reading of mirror-reversed orthography is powerful enough to reverse the flow of time in people’s mind.

The findings of Casasanto and Bottini (2014), together with those of other relevant studies, are very interesting. However, it is noteworthy that these aforementioned studies share one commonality, i.e., all languages under scrutiny are written unidirectionally. The question of whether such a causal effect of writing direction on temporal cognition can be found in speakers whose native languages adopt a bidirectional orthographical system remains underexplored in the literature. Given Casasanto and Bottini’s (2014) preliminary findings, we presuppose two kinds of possible logic relationship between the influence of standard bidirectional orthographies and that of mirror-reversed bidirectional orthographies on temporal thinking. If there is a causal effect of standard bidirectional orthographies of a certain language on people’s operation of mental timelines, then changing the orientations of bidirectional orthographies could reshape people’s representations of time in the opposite directions. On the contrary, if standard bidirectional orthographies of a certain language do not produce a causal effect on people’s construction of temporal cognition, then reversed bidirectional orthographies could not redirect the orientations of mental timelines as well.

The present study focused on Japanese which developed the bidirectional writing system early in the seventh century and preserved this usage until today (Insun, 2012). Two orientations are equally frequently employed in Japanese orthography, one proceeding HLR and one unfolding vertically from top to bottom (VTB). Therefore, Japanese speakers provide an optimal opportunity to examine the role of a bidirectional writing system in constructing people’s temporal cognition. Two experiments which recruited native speakers of Japanese as participants were performed to test our hypotheses. The tasks asked participants to process linguistic prime questions about time followed by nonlinguistic pictorial target stimuli about temporal relations. Experiment 1 sought to test whether two distinct writing orientations of a language could shape people’s representations of time via two discrepant spatial axes, while experiment 2 aimed to measure the effect of mirror-reversed bidirectional orthographies on the reconstruction of people’s mental timelines.

## Experiment 1

### Methods

#### Participants

Seventy-eight native speakers of Japanese (30 females, *M*age = 20.14, *SD*age = 0.97) from Nagoya City University (Nagoya, Japan) took part in this study in exchange for payment. All of them had normal or corrected-to-normal vision.

#### Ethics approval and consent

All procedures were approved by the ethics committee of Yangzhou University and written informed consent was obtained from all participants.

#### Materials

A set of 128 primes and 64 targets, all requiring true/false responses, was constructed.

##### Primes

**Fig. 1:**
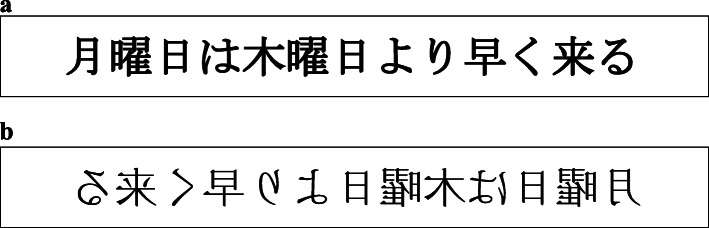
**a** An example of a horizontal standard-orthography prime. **b** An example of a horizontal mirror-orthography prime. The English equivalent for this Japanese sentence is “Monday comes earlier than Thursday”

**Fig. 2:**
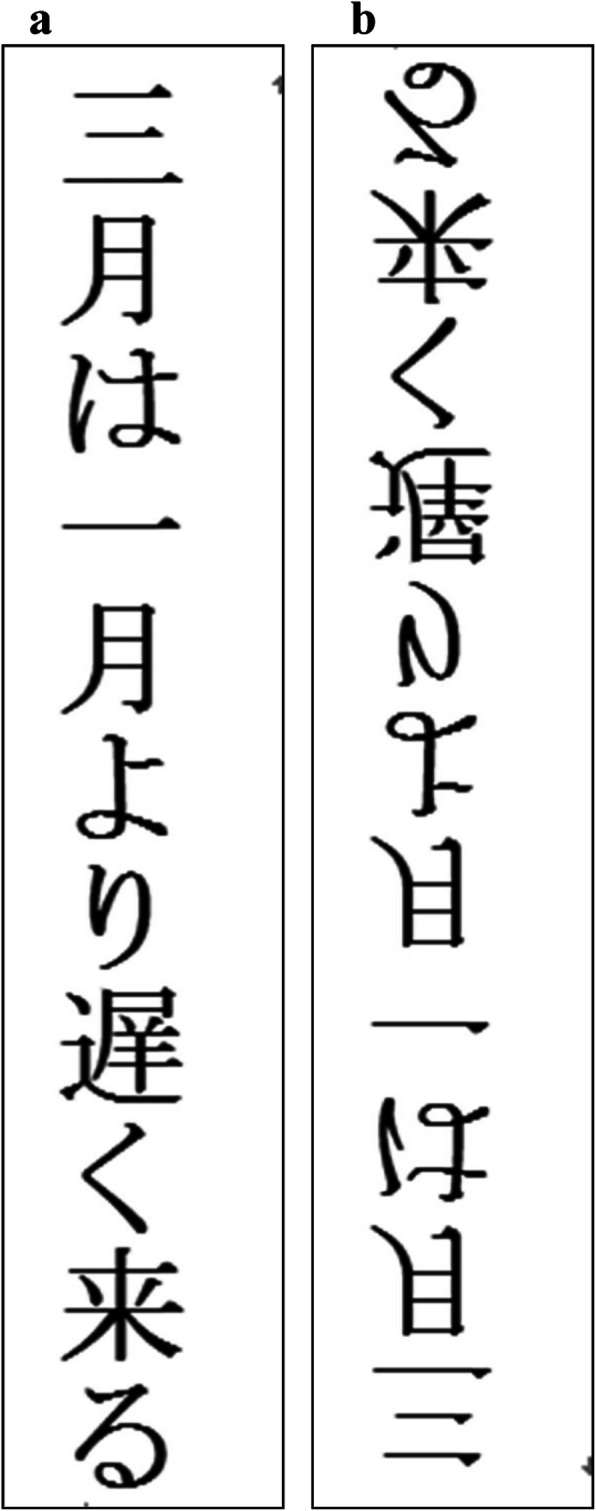
**a** An example of a vertical standard-orthography prime; **b** An example of a vertical mirror-orthography prime. The English equivalent for this Japanese sentence is “March comes later than January”

Sixty-four Japanese statements that used purely temporal terms “早/遅” (earlier/later) to describe the temporal relations were constructed. None of the sentences contained any spatial metaphoric expressions such as “front/back” to indicate the relationships between time units. The time units in the sentences included week days and months (e.g., 月曜日は木曜日より早く来る [English: “Monday comes earlier than Thursday], 三月は一月より遅く来る [English: “March comes later than January”]) (Appendix: Table 1). Participants were asked to verify whether the temporal order stated in the sentence was correct. Each prime contained one sentence, and all these statements were categorized into four types of primes: 32 horizontal standard-orthography primes in which each statement was written HLR, 32 horizontal mirror-orthography primes in which the same statements as in the horizontal standard-orthography primes were written HRL, 32 vertical standard-orthography primes in which each of the 32 remaining statements was arranged VTB, 32 vertical mirror-orthography primes in which the same statements as in the vertical standard-orthography primes were arranged vertically from bottom to top (VBT). Examples of the primes are shown in Figs. 1a, b, and 2a, b. In other words, each sentence appeared twice, once in the standard-orthography condition and once in the mirror-orthography condition of the same spatial axis, contributing to a total of 128 primes. Primes were equally often true and false.

##### Targets

**Fig. 3:**
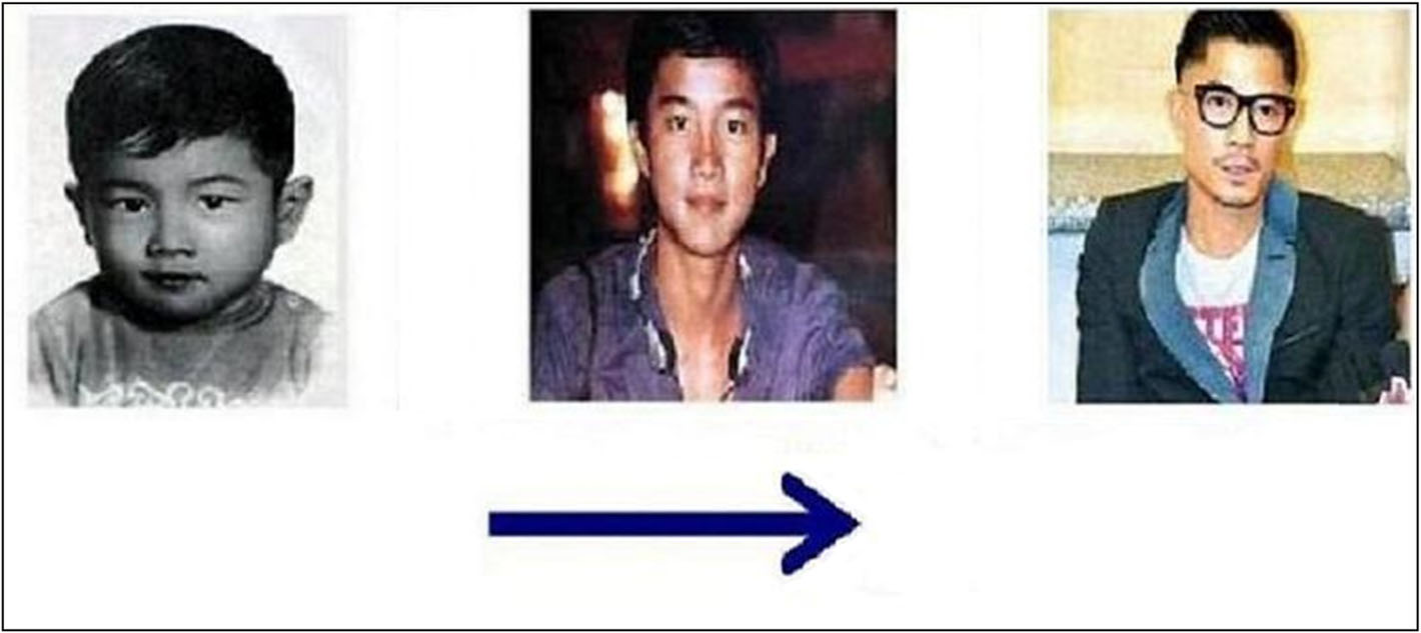
An example of a horizontal space-time compatible target. This is an instance of a true condition

**Fig. 4:**
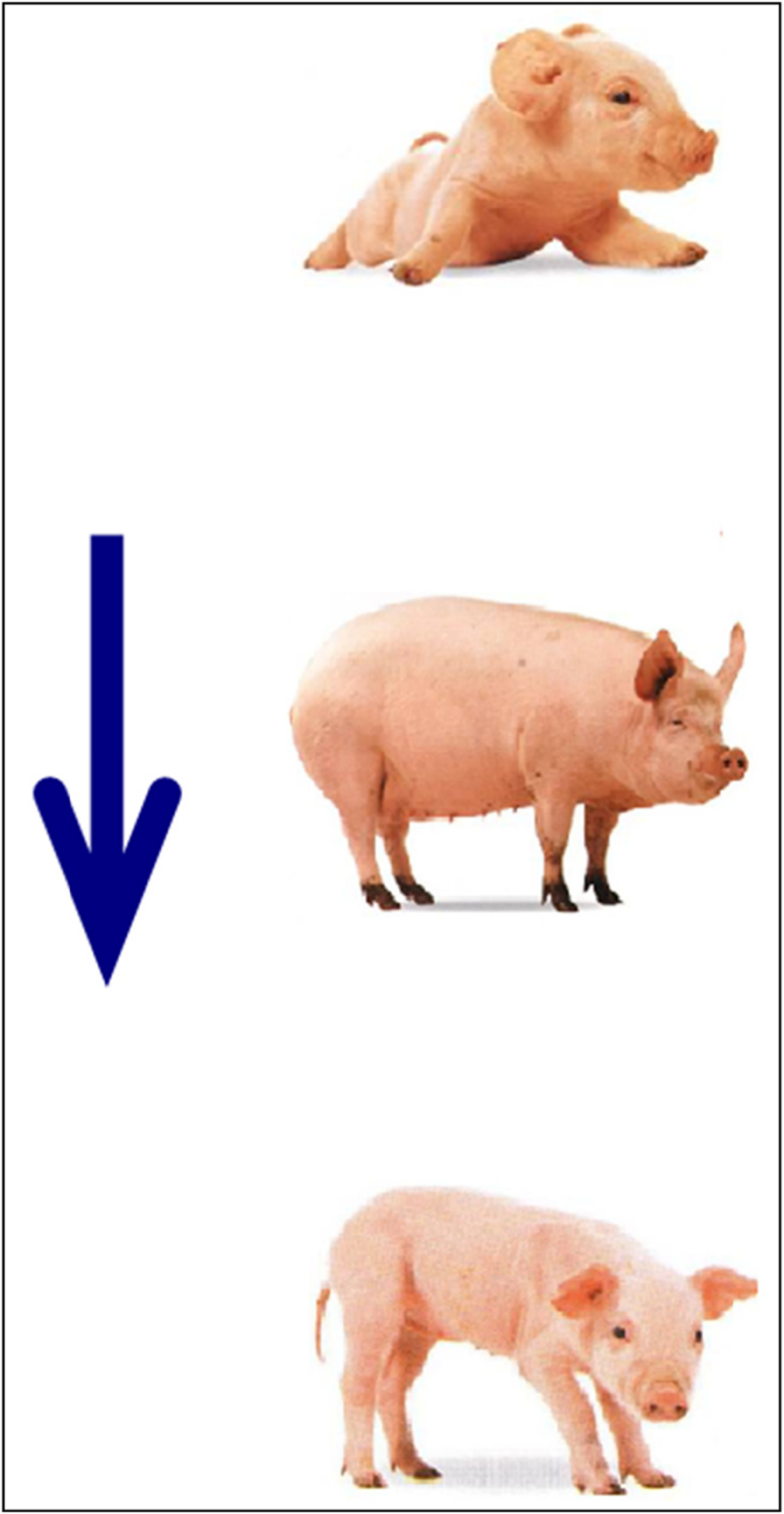
An example of a vertical space-time compatible target. This is an instance of a false condition

Targets comprised 64 triplets of pictures, all describing themes of temporal progression. Each triplet of pictorial stimulus (21.5 × 11.8 cm or 11.8 × 21.5 cm) showed a specific natural event at three different temporal stages (e.g., a film star aging, a pig growing, an orange tree growing). All these materials were categorized into two types of targets. Half were horizontal space-time compatible targets in which each triplet of pictorial stimulus was arranged from left to right as indicated by an arrow alongside the stimulus. Half were vertical space-time compatible targets in which each stimulus was arranged from top to bottom (see Figs. 3 and 4 as examples of the targets). In experiment 1, temporal sequences depicted in targets were “space-time compatible” because they unfolded in spatial axes compatible with the HLR or VTB orientation suggested by the standard orthographies of Japanese. Each target appeared twice, once after the standard-orthography prime and once the mirror-orthography prime of the same spatial axis. Target stimuli were equally often true and false. Participants had to judge if the temporal sequence described in the target was in the correct order according to the direction of the arrow (i.e., whether the 3 images were arranged in the correct temporal order, from the earliest to the latest).

##### Trials and blocks

Each participant completed four testing blocks, each comprising 32 trials. The four blocks were categorized according to the prime type: A horizontal standard-orthography block (each trial consisted of one horizontal standard-orthography prime followed by one pictorial target), a horizontal mirror-orthography block (each trial consisted of one horizontal mirror-orthography prime followed by one target), a vertical standard-orthography block (on each trial a vertical standard-orthography prime preceded a target), and a vertical mirror-orthography block (on each trial a vertical mirror-orthography prime preceded a target). The block order was counterbalanced across participants, and the orders of the trials were randomized within each block. The program started with four additional practice trials within each block, and the items used in the practice trials were not used in the subsequent experimental trials. The program did not allow participants to formally enter the testing trials until they achieved 100% accuracy in practice trials.

#### Procedures

All participants were tested individually in a quiet room, and all using the same desktop computer. On each trial, a fixation cross was presented in the center of the screen for 600 ms. Then, the prime appeared in the middle of the screen for 7000 ms. Participants were asked to verify whether the temporal order (earlier/later) stated in the sentence was correct, and they needed to respond as quickly and accurately as possible by pressing one of the two keys (the key “F” marked with a white sticker represented “False”; the key “J” marked with a red sticker represented “True”) on a standard keyboard. Upon entry of a response, the prime disappeared and the target appeared in the middle of the screen for 4000 ms. Participants were asked to judge if the temporal sequence described in the target was in the correct order according to the direction of the arrow by pressing one of the two keys referenced above. Once a judgment was made, a blank screen of 100 ms replaced the target and a new trial began.

#### Hypotheses

The purpose of using a priming paradigm in experiment 1 is to test whether Japanese speakers implicitly access space-time mappings commensurate with the bidirectional orthographies, i.e., (a) if earlier time points are automatically mapped onto the left side of space and later time points onto the right side along the horizontal axis; (b) if earlier time points are automatically mapped onto the top side and later time points onto the bottom side along the vertical axis. To be specific, if the processing of horizontal standard orthography can activate horizontal space-time congruity knowledge, Japanese speakers should be significantly faster to understand the space-time compatible target (i.e., a pictorial stimuli of a temporal sequence arranged HLR) after the standard-orthography prime (i.e., a sentence written HLR) than after the mirror-orthography prime (i.e., a sentence written HRL). In a similar vein, if the processing of vertical standard orthography can activate vertical space-time congruity knowledge, Japanese speakers should be significantly faster to handle the space-time compatible target (i.e., a pictorial stimuli of a temporal sequence arranged VTB) after the standard-orthography prime (i.e., a sentence written VTB) than after the mirror-orthography prime (i.e., a sentence written VBT).

### Data analysis and results

We analyzed participants’ responses to targets. Results from seven participants whose accuracy rates were egregious outliers (more than 10 *SD* away from the overall mean accuracy) were considered invalid and excluded from the dataset. Targets which recorded a response latency farther than 3 *SD* away from each participant’s mean respectively after the four types of primes (6.64%) and targets on which participants made errors (5.25%) were omitted from the RTs’ analyses.

**Fig. 5:**
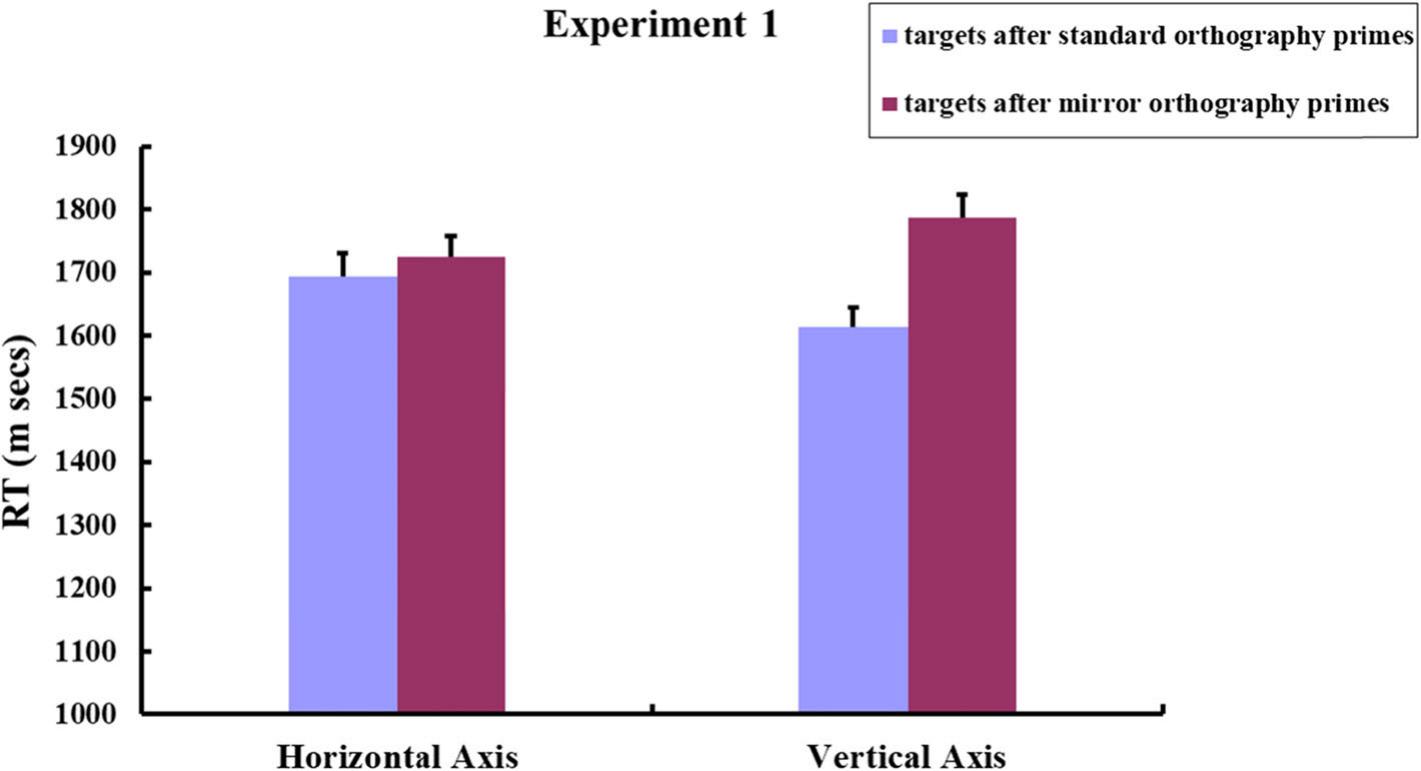
Experiment 1: Mean RTs for targets after the horizontal standard-orthography/mirror-orthography primes and the vertical standard-orthography/mirror-orthography primes by Japanese speakers. The figure plotted by participants’ mean RTs. Error bars indicate standard errors of the mean

The remaining response data were submitted to a by-participants 2 × 2 repeated measures ANOVA [Spatial Axis (horizontal, vertical) × Prime Type (targets after standard orthography primes, targets after mirror orthography primes)]. A by-items 2 × 2 ANOVA (spatial axis × prime type) was also computed. The results revealed a significant main effect of prime type [*F*1 (1, 70) *=* 33.26, *p* < 0.001, partial *η*^2^ = 0.322; *F*2 (1, 31) *=* 27.15, *p* < 0.001, partial η^2^ = 0.467], and a nonsignificant main effect of spatial axis [both *F*s < 1]. A significant spatial axis × prime type interaction was observed [*F*1 (1, 70) *=* 6.96, *p* < 0.05, partial *η*^2^ = 0.09; *F*2 (1, 31) *=* 6.296, *p* < 0.05, partial η^2^ = 0.169]. These results were not due to speed-accuracy trade-offs, because participants’ response accuracy for target items did not differ significantly [*F* < 1] across the four blocks (94.89%, 94.63%, 95.03% and 94.54% respectively). Planned paired *t* tests showed that participants responded to vertical targets significantly faster after vertical standard-orthography primes than after vertical mirror-orthography primes [*t*1 (70) = −5.48, *P* < 0.001, *d* = 0.65; *t*2 (31) = −4.25, *P* < 0.001, *d* = 0.75], but they responded to horizontal targets just as quickly after standard-orthography primes as after mirror-orthography primes along the horizontal axis [*t*1 (70) = −0.915, *p* = 0.363; *t*2 (31) = −0.945, *p* = 0.352]. Figure 5 plotted the mean RTs for targets respectively following different types of primes.

Participants’ correct responses to primes were additionally checked by paired *t* tests, and results showed that the response patterns for primes were consistent with those for targets. Participants responded to vertical standard-orthography primes significantly faster than to vertical mirror-orthography primes [*t*1 (70) = −4.43, *P* < 0.001, *d* = −0.53; *t*2 (31) = −6.27, *P* < 0.001, *d* = −1.11], but they responded to standard-orthography primes almost as quickly as mirror-orthography primes along the horizontal axis [*t*1 (70) = −1.16, *p* = 0.25; *t*2 (31) = −1.33, *p* = 0.195].

## Discussion

While Japanese speakers did show vertical space-time congruity priming effect, their RTs for space-time congruity targets were almost equally fast after the standard-orthography primes and the mirror-orthography primes along the horizontal axis. The results indicated that even the online processing of sentences written in standard horizontal orthography may not trigger the corresponding space-time associations. Therefore, the results of experiment 1 could only partially explicate the impact of bidirectional writing/reading patterns on people’s spatial layout of mental timelines. For one thing, Japanese speakers’ vertical space-time mappings were consistent with the conventions of standard VTB Japanese writing direction. They responded to VTB arranged target stimuli of temporal sequences significantly faster after standard VTB orthography primes than after mirror VBT orthography primes, suggesting that Japanese speakers possess a VTB timeline in his/her mind. For another, Japanese speakers did not appear to represent elapsing time in the same HLR direction as they read and write. They responded to HLR arranged target stimuli almost as fast after standard HLR orthography primes as after mirror HRL orthography primes, indicating that Japanese speakers did not favor a HLR oriented space-time association.

## Experiment 2

Our attempt to validate the causal effect of the bidirectional writing/reading system on the construction of people’s mental timelines turned out to be simultaneously a success and a failure in experiment 1. While moving one’s eyes (or one’s attention) through space and time in the standard vertical Japanese orthographical direction during reading did cause Japanese speakers to access the corresponding conceptions of timelines, no orthography-dependent modulation of the horizontal spatial-temporal mental representations was identified in Japanese speakers. In view of the results from experiment 1, we assume that changing the orientations of orthographies could activate the reversed vertical space-time mappings, but could not restructure the horizontal representations of time in the opposite direction. Experiment 2 was designed to test such hypotheses. Prior to experiment 2, a new group of Japanese participants received a short training of reading Japanese texts written in mirror-reversed orthographies. During the experiment, we exposed these participants to instructions presented in mirror-reversed orthographies and to space-time incompatible targets. In experiment 2, targets were “space-time incompatible” because pictorial stimuli of temporal sequences were arranged HRL or VBT, inconsistent with either of the two spatial orientations dictated by standard orthographies of Japanese but congruent with the two directions of mirror-reversed orthographies.

### Methods

#### Participants

Sixty-six native speakers of Japanese (35 females, *M*age = 20.65, *SD*age = 1.39) from Saitama University (Saitama, Japan) took part in this study in exchange for payment. All of them had normal or corrected-to-normal vision. Prior to experiment 2, participants were trained to read Japanese texts written in mirror-reversed orthographies for about 20 min. For each participant, he/she read a similar mix of texts arranged HRL and VBT, i.e., approximately half of the texts were written HRL and half VBT.

#### Ethics approval and consent

All procedures were approved by the ethics committee of Yangzhou University and written informed consent was obtained from all participants.

#### Materials and procedures

**Fig. 6:**
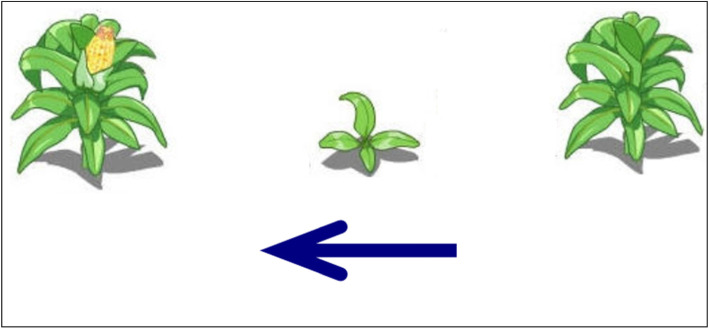
An example of a horizontal space-time incompatible target. This is an instance of a false condition

**Fig. 7:**
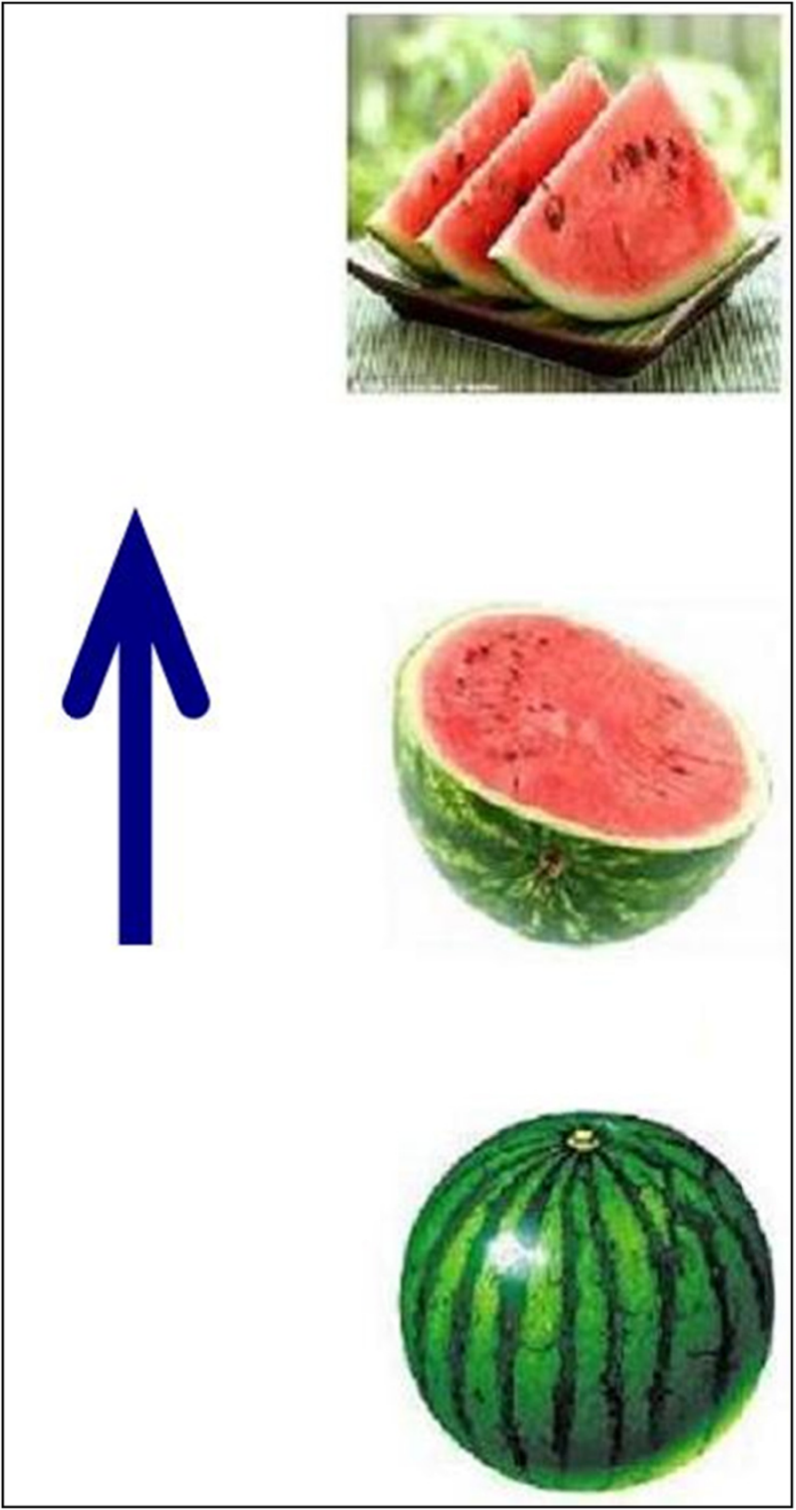
An example of a vertical space-time incompatible target. This is an instance of a true condition

All materials and procedures were identical to experiment 1 with the following exceptions. First, the pictorial stimuli used for targets were categorized into two types. Half were horizontal space-time incompatible targets in which each triplet of pictorial stimulus was arranged from right to left as indicated by an arrow alongside the stimulus. Half were vertical space-time incompatible targets in which each stimulus was arranged from bottom to top (see Figs. 6 and 7 as examples of the targets). Second, instructions were presented in mirror-reversed orthographies, i.e., approximately half of the texts were written HRL and half were VBT for each participant (examples of the mirror-reversed orthographies can be seen in Figs. 1b and 2b).

#### Hypotheses

As outlined above, since the processing of horizontal mirror orthography may not activate horizontal space-time incongruity knowledge, Japanese speakers should understand the space-time incompatible target (i.e., a pictorial stimuli of a temporal sequence arranged HRL) equally fast after the standard-orthography prime (i.e., a sentence written HLR) and after the mirror-orthography prime (i.e., a sentence written HRL). On the contrary, given that the processing of vertical mirror-orthography may activate vertical space-time incongruity knowledge, Japanese speakers should be significantly faster to handle the space-time incompatible target (i.e., a pictorial stimuli of a temporal sequence arranged VBT) after the mirror-orthography prime (i.e., a sentence written VBT) than after the standard-orthography prime (i.e., a sentence written VTB).

### Data analysis and results

We analyzed participants’ responses to targets. One participant failed to complete experiment 2 because of unexpected bugs in computer programs. Moreover, results from six participants whose accuracy rates were egregious outliers (more than 10 *SD* away from the overall mean accuracy) were considered invalid and excluded from the dataset. Targets which recorded a response latency farther than 3 *SD* away from each participant’s mean respectively after the four types of primes (6.22%) and targets on which participants made errors (4.29%) were omitted from the RTs’ analyses.

**Fig. 8:**
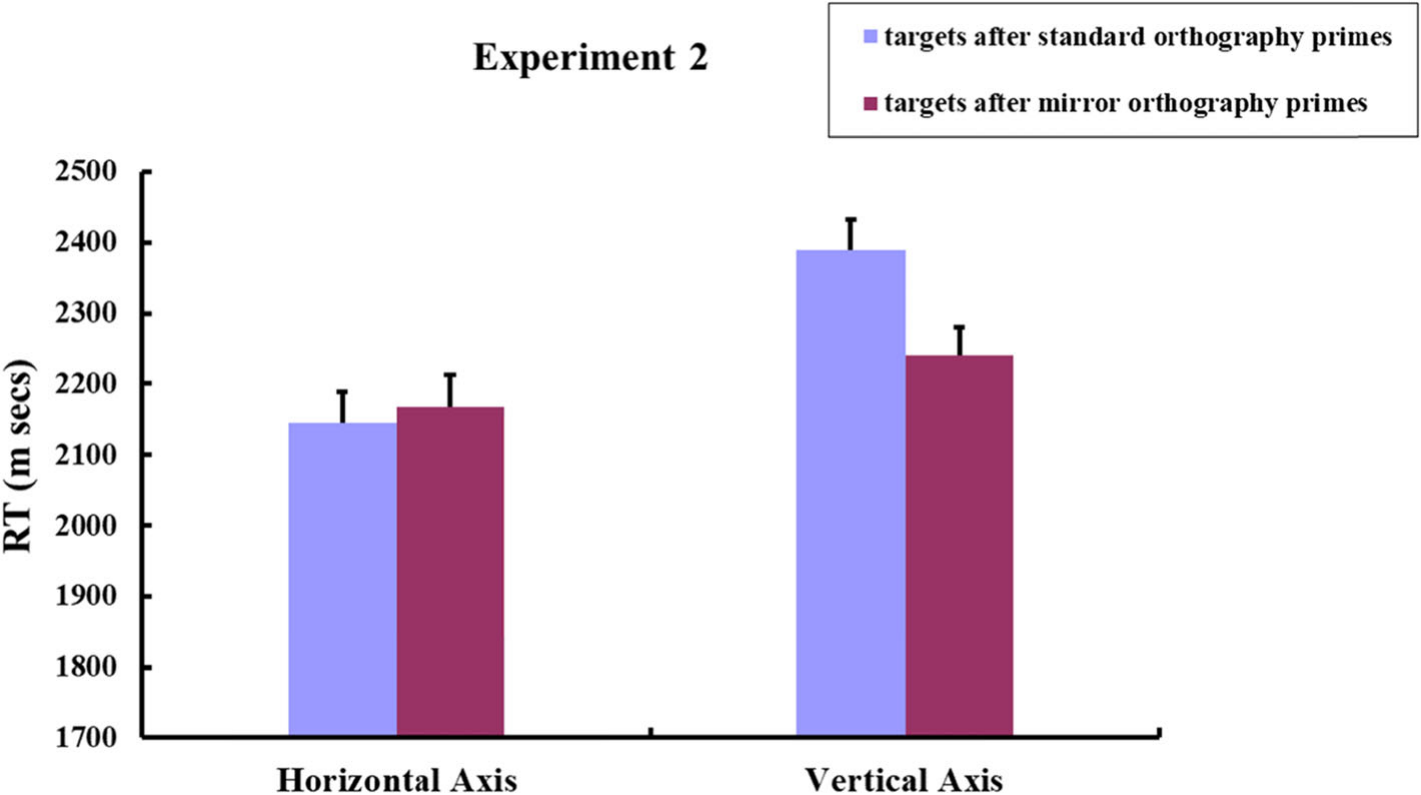
Experiment 2: Mean RTs for targets after the horizontal standard-orthography/mirror-orthography primes and the vertical standard-orthography/mirror-orthography primes by Japanese speakers. The figure plotted by participants’ mean RTs. Error bars indicate standard errors of the mean

The remaining response data were submitted to a by-participants 2 × 2 repeated measures ANOVA [spatial axis (horizontal, vertical) × prime type (targets after standard orthography primes, targets after mirror orthography primes)]. A by-items 2 × 2 ANOVA (spatial axis × prime type) was also computed. The results revealed significant main effects of spatial axis [*F*1 (1, 58) *=* 100.04, *p* < 0.001, partial *η*^2^ = 0.633; *F*2 (1, 31) *=* 74.71, *p* < 0.001, partial *η*^2^ = 0.707], and prime type [*F*1 (1, 58) *=* 5.79, *p* < 0.05, partial *η*^2^ = 0.091; *F*2 (1, 31) *=* 4.98, *p* < 0.05, partial *η*^2^ = 0.136]. A significant spatial axis × prime type interaction was observed [*F*1 (1, 58) *=* 32.25, *p* < 0.001, partial *η*^2^ = 0.357; *F*2 (1, 31) *=* 24.50, *p* < 0.001, partial *η*^2^ = 0.441]. These results were not due to speed-accuracy trade-offs, because participants’ response accuracy for target items did not differ significantly [*F* < 1] across the four blocks (95.18%, 96.35%, 95.97%, 95.45% respectively). Planned paired *t* tests showed that participants responded to vertical targets significantly faster after vertical mirror-orthography primes than after vertical standard-orthography primes [*t*1 (58) = −5.67, *P* < 0.001, *d* = −0.74; *t*2 (31) = −3.53, *P* < 0.01, *d* = −0.62], but they responded to horizontal targets just as quickly after standard-orthography primes as after mirror-orthography primes along the horizontal axis [*t*1 (58) = −0.68, *p* = 0.50; *t*2 (31) = −0.66, *p* = 0.515]. Figure 8 plotted the mean RTs for targets respectively following different types of primes.

We additionally performed paired *t* tests to analyze participants’ correct responses to primes, and results indicated that the response biases for primes were in line with those for targets. Participants responded to vertical mirror-orthography primes significantly faster than to vertical standard-orthography primes [*t*1 (58) = −2.18, *P* < 0.05, *d* = −0.28; *t*2 (31) = −5.07, *P* < 0.001, *d* = −0.89], but they responded to standard-orthography primes just as quickly as mirror-orthography primes along the horizontal axis [*t*1 (58) = −0.58, *p* = 0.57; *t*2 (31) = −1.10, *p* = 0.28]. These results corroborated that while the training of mirror reading did not appear to affect Japanese speakers’ horizontal reading habits, it did (at least temporarily) reverse their reading habits along the vertical axis which accordingly redirected the orientation of the vertical mental timeline.

An extra question pertinent to the experimental design of this study which may warrant clarification is that temporal sequences depicted in the target pictorial stimuli spanned a large range of durations: from events that lasted only seconds or minutes (e.g., an apple being eaten) to intervals that spanned years or decades (e.g., a person at different stages in life). To examine if the duration of time intervals presented in the targets affect participants’ performance, we conducted by-items paired *t* tests comparing RTs on targets that depicted long-time interval and those depicted short intervals in each block. Results confirmed the non-significant difference in participants’ responses to targets that represented long and short temporal durations within the same testing block. In experiment 1, the results for each of the four blocks are (the four blocks were simply referred to as block 1, 2, 3 and 4): [block 1: *t* (15) = 1.71, *p* = 0.107; block 2: *t* (15) = 1.52, *p* = 0.149; block 3: *t* (15) = −0.60, *p* = 0.557; block 4: *t* (15) = 0.59, *p* = 0.562]. In experiment 2, the results are [block 1: *t* (15) = −1.72, *p* = 0.106; block 2: *t* (15) = 0.83, *p* = 0.419; block 3: *t* (15) = 1.66, *p* = 0.118; block 4: *t* (15) = -0.92, *p* = 0.372].

## Discussion

As predicted, the results of experiment 2 confirmed both the existence of Japanese speakers’ vertical space-time incongruity priming effect and the nonexistence of horizontal space-time incongruity priming effect. Comparing RTs for space-time incongruity targets after the standard-orthography primes and those after the mirror-orthography primes revealed significant differences along the vertical axis and nonsignificant difference along the horizontal axis. Mirror reading of bidirectional orthographies therefore reversed the vertical timeline in the mind but did not modulate horizontal spatial-temporal mental schemas.

It is noteworthy that stimuli and procedures employed in the present research are not the same as the temporal congruency categorization paradigm used by a number of relevant studies (e.g., Boroditsky et al., 2011; Casasanto and Bottini, 2014; Ouellet et al., 2010; Santiago et al., 2010; Vallesi et al., 2014). These studies measured participants’ space-time mappings via the array of response keys, given that the stimuli did not convey any information about spatiotemporal associations but participants were asked to press horizontally (HLR/HRL) or vertically (VTB/VBT) arranged keys (one key representing “past” and the other “future”) which designated the directions of time flow. Therefore, participants categorized future or past time point through key arrangement which was the most important variable under investigation. On the contrary, our research examined participants’ space-time associations via the setup of stimuli, because the response keys did not reveal any spatiotemporal messages (one key representing “true” and the other “false”) but the linguistic prime stimuli and pictorial target stimuli were arranged horizontally or vertically to indicate the linear path of elapsing time. The experimental paradigm abided by such a design principle has been used to successfully examine spatiotemporal representation in relevant studies (e.g., Boroditsky, 2001; Tse & Altarriba, 2008; Yang & Sun, 2016b). In a word, the critical design for the present experiment lies in the arrangement of stimuli rather than response keys. Which of the two experimental paradigms could better capture the cognitive mechanisms of temporal processing remains an unexplored issue, as different investigators may favor one paradigm over the other. What is clear thus far, however, is that the stimuli and procedural distinction does not constitute a potential factor that affects the spatiotemporal compatible/incompatible effects observed.

## Conclusion

The present study recruited Japanese speakers as participants to examine whether parallel use of two distinct writing/reading directionalities could shape people’s mental representations of time and whether changing the orientations of the bidirectional orthographies could lead to corresponding reversals in people’s implicit mental timelines. The experimental setups asked participants to process horizontal/vertical standard-orthography and mirror-orthography linguistic primes followed by horizontal/vertical space-time compatible or incompatible non-linguistic targets. Across two experiments, Japanese speakers showed both space-time congruity (experiment 1) and space-time incongruity (experiment 2) priming effect along the vertical axis, but no such priming effects were found along the horizontal axis. The evidence demonstrated that Japanese speakers encoded passage of time into a top-to-bottom linear path commensurate with the VTB writing direction, but they did not align their mental representations of time with the HLR writing orientation. Accordingly, exposure to mirror-reversed bidirectional orthographies resulted in changes in Japanese speakers’ vertical but not horizontal space-time mappings.

Results of experiment 1 suggest that two distinct writing orientations of a particular language could not bring about two coexisting mental timelines. However, such findings diverge from those of Casasanto and Bottini (2014) and many other existing observations which established the causal role of a unidirectional writing/reading orientation in shaping how people represent time spatially (e.g., Bergen & Chan Lau, 2012; Ding et al., 2015; Fuhrman & Boroditsky, 2010; Ouellet et al., 2010; Santiago et al., 2010; Tversky et al., 1991; Vallesi et al., 2014). Experiment 1 of the present study clarifies previous findings by highlighting the fact that the relationship between bidirectional orthographies and mental representations of time is a much more complicated issue, as bidirectional orthographies of a certain language (e.g., HLR and VTB for Japanese) may not simultaneously produce such a crucial impact akin to that of a unidirectional orthography on temporal cognition. According to the existing literature, there is hardly any study involving a sample of speakers whose native language followed a bidirectional writing system, with the exception of de Sousa (2012). Via a card arrangement task and a time-pointing task that study yielded results somewhat similar to experiment 1 of the present research. The language under investigation in de Sousa (2012) is Cantonese which employed a mixture of HLR, VTB, and HRL writing directions, with HLR being the relatively dominant one. However, a Cantonese speaker was not found to accommodate bidirectional or even multidirectional mental timelines in his/her mind. Rather, each individual represented time via a single and unidirectional spatial axis. While older people displayed a salient propensity to think about time HRL, younger people predominately accessed a timeline-oriented HLR.

Vallesi et al. (2014) tentatively put forward the competition hypothesis which might provide one of the plausible explanations for the observed association (and dissociation) between bidirectional orthographies and mental representations of time in experiment 1. This hypothesis proposes that hybrid writing/reading habits might compete with each other for cognitive resources in people’s conceptual system, and thus attenuate or eliminate the causal effect of either (or even both) of the writing/reading directions on space-time mappings. Although the validity of the competition hypothesis is pending for further examinations, the evidence procured thus far can in principle collapse the decisive role of HLR writing/reading orientation in shaping Japanese speakers’ mental construals of timelines.

While the competition hypothesis may logically stand, results of experiment 1 give rise to another interesting question: Why Japanese speakers overwhelmingly access a VTB timeline rather than a HLR space-time mapping in the mind? Two potential possibilities may presumably account for this finding. One possibility points to the orientation/sequence of lines and columns in Japanese writing. Note that HLR writing direction for Japanese (and for all the languages under scrutiny in previous studies) exclusively refers to characters or words written in that orientation within a single row, whereas VTB refers to the writing direction within a single column. If the text exceeds one line or one column, both HLR and VTB orthography will comprise an additional spatial dimension. For HLR writing orientation, when the end of a line is reached, words or characters begin on a new line which is placed below the preceding one. For Japanese VTB writing, when texts reached the end of a column, the next column is written to the left of the previous one, suggestive of a HRL sequence of columns. Notably, the HLR system for characters/words coexist with a VTB sequence of lines, with former lines on top and latter lines below. Therefore, even if Japanese speakers are writing/reading via the HLR system, they still have to follow the rules of the VTB sequence of lines. This kind of orthographical constraint, together with the VTB writing system itself, may remarkably reinforce the VTB space-mapping. In the meantime, the HRL sequence of columns for the VTB writing system may attenuate or even counteract the effect generated by the HLR writing system. A second possibility concerns the metaphoric patterns for time in Japanese language. Japanese use both sagittal front/back [e.g., 一昨日 (the day before yesterday), 明後日 (the day after tomorrow)], and vertical top/bottom [e.g., 先週 (last week), 来月 (next month)] spatiotemporal metaphors to talk about time. However, there are no horizontal left/right terms to express temporal information. The existence of vertical metaphors for time and the absence of horizontal metaphors may also contribute to Japanese speakers’ significant bias to the present time via a VTB spatial axis. Though the two possibilities elucidated above are yet to be comprehensively validated by experimental evidence, the claim that bidirectional orthographies themselves can both serve as the potent sources for space-time associations is regretfully untenable.

Casasanto and Bottini (2014) found that native Dutch speakers represented time flow in terms of a rightward-directed mental linear path, consistent with the unidirectional HLR writing orientation of Dutch. Furthermore, 5 min of reading mirror-reversed texts completely changed the directionality of Dutch speakers’ mental timelines established on the daily basis of reading/writing experience. Casasanto and Bottini (2014) accounted for this phenomenon in terms of the representational flexibility of time, according to which culture-specific space-time mappings would be conditioned by the relationship between space and time in the immediate context of the physical world. Results yielded from experiment 2 of the current study partially corroborated Casasanto and Bottini (2014)’s interpretations, given Japanese speakers’ representational flexibility of vertical mental timeline. Nevertheless, the results of experiment 2 also contradict Casasanto and Bottini’s (2014) finding by manifesting that mirror reading of HLR orthography does not modify spatial representations of time in Japanese speakers. We assume that exposure to a new orthography opposite to the direction of the standard orthography in people’s native languages may possibly change the orientation of the mental timeline within minutes, as maintained by Casasanto and Bottini (2014). This kind of representational flexibility in people’s conceptual system may be readily generalized to speakers of those languages which are written unidirectionally. However, generalization of such a hypothesis to speakers whose native languages adopt bidirectional orthographies should be treated with caution. Our work suggests that the (non) existence of representational flexibility depends on the relative strength of the two orthographical orientations of a certain language which may contribute to the construction of temporal cognition. On the basis of our empirical data and the competition hypothesis (Vallesi et al., 2014), we propose two possibilities which serve to extend and revise Casasanto and Bottini (2014)’s position. First, only when two writing orientations of a certain language both enact determinant roles in shaping speakers’ representations of time may mirror-bidirectional orthographies simultaneously reverse the two timelines in the mind. Second, if either of the two writing directions itself is unable to shape a mental timeline, exposure to the corresponding mirror-reversed orthography will accordingly not drive such an effect, namely, representational flexibility of time.

There is another interesting issue emerging from this study that remains unaddressed: Why Japanese speakers temporality changed their reading habits along the vertical axis after a short period of reading training in experiment 2 (as suggested by significantly faster RTs in the vertical mirror-orthography primes than in the vertical standard-orthography primes), while they showed no significant difference in RTs between the horizontal standard-orthography primes and the horizontal mirror-orthography primes? The present results along the horizontal axis were consistent with some of the previous findings which indicate that native speakers of East Asian languages (e.g., Chinese, Japanese, and Korean) understood horizontal standard-orthography texts and mirror-orthography texts written in their native languages almost equally fast (Kess & Miyamoto, 1999; Ren, 2010; Zhou et al., 2008). However, none of the existing studies, to the best of our knowledge, directly probed into response latencies and accuracies between the possessing of vertical standard-orthography texts and that of vertical mirror-orthography texts by Japanese speakers. We assume that current results about the vertical primes might point to the mechanisms underlying the processing of individual kanji (Chinese characters) within a line or column of Japanese texts.

Japanese employed numerous kanji borrowed from the Chinese logographic writing system early in the seventh century and preserved their active usage until today (Kess & Miyamoto, 1999). Unlike alphabetic languages (e.g., English), the basic meaningful units in logographic languages such as Chinese and Japanese are kanji or characters (Chen, 1996; Li & Chen, 1997). Over 90% of kanji are composed of sub-character constituent units, i.e., recurring integral stroke patterns called radical and stem (Chen, 1996). The remaining kanji (less than 10%), without radical or stem, belong to the category named independent structure. The radical (the semantic component) provides a cue as to the meaning of the whole character, while the stem (the phonetic component) usually indicate the pronunciation of the character (Fang & Wu, 1989). A combination of a radical and a stem typically form a horizontal left-right (with radical on the left and stem on the right, and vice versa) or vertical top-bottom (e.g., with radical on the top and stem on the bottom) structure layout of a kanji. A wealth of experimental research demonstrates that kanji recognition is a basic process in Chinese and Japanese reading, and radicals, the most fundamental functional and orthographic units of kanji, play a primary or even decisive role in the identification of a kanji (Chen et al., 1996; Feldman & Siok, 1997; Leong & Tamaoka, 1998; Li & Chen, 1997; Taft et al., 1999; Tong & Yip, 2015; Wu et al., 2012; Zhou et al., 2013). Prior to the holistic processing of kanji, people almost unanimously do analytic processing of radicals first. Especially in the early stages of processing, kanji are probably perceived as radical components rather than as whole characters. The effective activation of the graphemic and semantic information at the radical level can significantly facilitate the recognition of the entire kanji (Chen et al., 1996; Feldman & Siok, 1997; Li & Chen, 1997; Liu et al., 2010; Taft et al., 1999; Tong & Yip, 2015; Zhou et al., 2013).

Note that the magnitude of the effects of radical level recognition on processing the whole kanji may be variant, depending on such crucial factors as the radical position (Chi et al., 2014; Feldman & Siok, 1997; Li & Chen, 1997; Tong & Yip, 2015; Tsang & Chen, 2009). Previous studies revealed a dominant effect of the lower part in the recognition of top-bottom structured kanji (Chi et al., 2014; Gao & Xiao, 2017). To be specific, those kanji with radicals on the bottom side were processed faster than kanji with the same radicals which were, on the contrary, placed on the top. However, such a bias in RTs did not exist for understanding left-right structured kanji, irrespective of whether radicals were located on the left or right (Chi et al., 2014; Feldman & Siok, 1997; Yin, 2016). These findings could presumably explain the phenomenon that we observed in the present study. The experimental materials of the standard orthography primes contained independent structured kanji without radicals (e.g., 月, 日), left-right (e.g., 遅, 曜), and top-bottom (e.g., 金, 早, 来) structured kanji, with radicals either on the left or on the top side. In the horizontal mirror-reversed condition, those original kanji with radicals on the left now moved the radicals to the right side, while the relative position of the radicals on the top remained unchanged in the top-bottom structured kanji. Therefore, it is unsurprising that participants responded to standard-orthography primes and mirror-orthography primes almost equally fast along the horizontal axis, as their processing speed for individual kanji did not differ significantly across the two conditions. Meanwhile, it is obvious that both the left-right and top-bottom structured kanji reversed their relative positions of radicals in the vertical mirror-reversed condition. Those original kanji with radicals on the top now moved the radicals to the bottom side. As indicated above, the reversal of left-right structured kanji, in comparison with the standard left-right structured kanji, may not produce a distinct effect for processing speed. But the redirection of top-bottom structured kanji can affect RTs for recognizing kanji. Though the artificial reversed kanji is not a “genuine” orthography, 20 min of mirror reading training may be sufficient enough to familiarize participants with the new orthographical system which can accordingly trigger and intensify the dominant effect of the lower part in recognizing top-bottom structured kanji, i.e., RTs for understanding reversed top-bottom kanji (with radicals on the bottom) is significantly shorter than that for standard top-bottom kanji (with radicals on the top). Faster speed for processing individual top-bottom kanji in the vertical mirror-reversed prime condition may eventually contribute to shorter RTs for understanding the entire column of the text. A more significant issue that warrants clarification is that all the characters in the primes (including both the Japanese characters and Chinese characters) are themselves upside-down, irrespective of whether the character contains a radical or where the radical is positioned. This may also explain why the significant difference in response latencies only existed between vertical standard-orthography primes and vertical mirror-orthography primes, while no significant difference in RTs was found between the standard-orthography and mirror-orthography prime conditions along the horizontal axis.

Finally, we can not thoroughly rule out the possibility that the effects found in the present study and in Casasanto and Bottini (2014) might have to do with the level of competence in performing a given task as opposed to any stable mental representation of temporal phenomena that the participants may possess. For one thing, in experiment 2 participants responded at almost the same speed to horizontal linguistic primes in both the standard and mirror-reversed directions. This suggests that the reading training did not appear to affect participants’ competence in processing the prime stimuli, and thus there may be little reason to expect that participants’ responses to prime stimuli can influence their competence in understanding the target stimuli. For another, regardless of the discrepancies in content, there was internal consistency between the prime stimuli and the target stimuli, as they had similar form. They involved reading either linguistic or pictorial symbols arranged in a row or a column in a given dimension, and participants had been trained to read in that direction within a limited period of time. Participants’ propensity to represent time along the horizontal axis, even if it existed, may not be stable and significant enough to be explicitly activated by the experimental setups in the task. Therefore, the conclusions drawn and the underlying explanations elaborated in the paper, although highly plausible, should be treated with caution and call for further examinations.

## Data Availability

The data and materials of this article are available by request to the corresponding author.

## Appendix

Original Japanese sentences used for the experimental materials and their English translations

**Table 1:** The left column contains the original Japanese sentences and the right column their English translations

Japanese	English translations
三月は一月より遅く来る	March comes later than January
火曜日は水曜日より早く来る	Tuesday comes earlier than Wednesday
六月は五月より早く来る	June comes earlier than May
土曜日は火曜日より早く来る	Saturday comes earlier than Tuesday
十月は十二月より早く来る	October comes earlier than December
土曜日は金曜日より遅く来る	Saturday comes later than Friday
月曜日は木曜日より遅く来る	Monday comes later than Thursday
十月は八月より早く来る	October comes earlier than August
木曜日は火曜日より遅く来る	Thursday comes later than Tuesday
九月は八月より遅く来る	September comes later than August
土曜日は木曜日より早く来る	Saturday comes earlier than Thursday
四月は二月より遅く来る	April comes later than February
十一月は六月より早く来る	November comes earlier than June
火曜日は土曜日より早く来る	Tuesday comes earlier than Saturday
十月は一月より早く来る	October comes earlier than January
水曜日は金曜日より遅く来る	Wednesday comes later than Friday
火曜日は木曜日より早く来る	Tuesday comes earlier than Thursday
九月は十月より遅く来る	September comes later than October
土曜日は水曜日より早く来る	Saturday comes earlier than Wednesday
金曜日は木曜日より遅く来る	Friday comes later than Thursday
月曜日は火曜日より遅く来る	Monday comes later than Tuesday
十一月は四月より遅く来る	November comes later than April
五月は九月より早く来る	May comes earlier than September
七月は八月より遅く来る	July comes later than August
四月は五月より早く来る	April comes earlier than May
月曜日は木曜日より早く来る	Monday comes earlier than Thursday
八月は十二月より遅く来る	August comes later than December
土曜日は金曜日より早く来る	Saturday comes earlier than Friday
水曜日は火曜日より遅く来る	Wednesday comes later than Tuesday
三月は五月より遅く来る	March comes later than May
火曜日は金曜日より遅く来る	Tuesday comes later than Friday
一月は二月より早く来る	January comes earlier than February

## Abbreviations

HLR: Horizontally from left to right
HRL: Horizontally from right to left
VTB: Vertically from top to bottom
VBT: Vertically from bottom to top

## References

[CR1] Bergen BK, Chan Lau TT (2012). Writing direction affects how people map space onto time. Frontiers in Psychology.

[CR2] Boroditsky L (2000). Metaphoric structuring: Understanding time through spatial metaphors. Cognition.

[CR3] Boroditsky L (2001). Does language shape thought? English and Mandarin speakers’ conceptions of time. Cognitive Psychology.

[CR4] Boroditsky, L., Fuhrman, O., & McCormick, K. (2011). Do English and Mandarin speakers think about time differently? *Cognition, 118*, 123–129. 10.1016/j.cognition.2010.09.010. 10.1016/j.cognition.2010.09.010 21030013

[CR5] Casasanto D, Lewandowska-Tomaszczyk B (2016). Temporal language and temporal thinking may not go hand in hand. *Conceptualizations of time*.

[CR6] Casasanto D, Bottini R (2014). Mirror reading can reverse the flow of time. Journal of Experimental Psychology: General.

[CR7] Chen HC, Bond MH (1996). Chinese reading and comprehension: A cognitive psychology perspective. *The handbook of Chinese psychology*.

[CR8] Chen YP, Allport DA, Marshall JC (1996). What are the functional orthographic units in Chinese word recognition: The stroke or the stroke pattern. The Quarterly Journal of Experimental Psychology.

[CR9] Chi H, Yan G, Xu X, Xia Y, Cui L, Bai X (2014). The effect of phonetic radicals on identification of Chinese phonograms: Evidence from eye movement. Acta Psychologica Sinica.

[CR10] de Sousa H (2012). Generational differences in the orientation of time in Cantonese speakers as a function of changes in the direction of Chinese writing. Frontiers in Psychology.

[CR11] Ding X, Cheng X, Fan Z, Liu H (2015). Is elapsing time really recoded into spatial linear representation in working memory?. Experimental Psychology.

[CR12] Fang SP, Wu P (1989). Illusory conjunctions in the perception of Chinese characters. Journal of Experimental Psychology: Human Perception and Performance.

[CR13] Feldman LB, Siok WWT (1997). The role of component function in visual recognition of Chinese character. Journal of Experimental Psychology Learning Memory and Cognition.

[CR14] Fuhrman O, Boroditsky L (2010). Cross-cultural differences in mental representations of time: Evidence from an implicit nonlinguistic task. Cognitive Science.

[CR15] Fuhrman O, McCormick K, Chen E, Jiang H, Shu D, Mao S, Boroditsky L (2011). How linguistic and cultural forces shape conceptions of time: English and Mandarin time in 3D. Cognitive Science.

[CR16] Gao R, Xiao C, Han B (2017). The dominant effect of lower part in Chinese characters recognition. *Proceedings of the 20*^*th*^*National Academic Congress of Psychology*.

[CR17] Insun. (2012). Japanese: beautiful and complex writing systems. Retrieved from http://www.topys.cn/article/detail?id=7600

[CR18] Kess JF, Miyamoto T (1999). *The Japanese mental lexicon: Psycholinguistic studies of kana and kanji processing*.

[CR19] Lakoff G, Johnson M (1980). *Metaphors we live by*.

[CR20] Lakoff G, Johnson M (1999). *Philosophy in the flesh: The embodied mind and its challenge to Western thought*.

[CR21] Leong CK, Tamaoka K (1998). *Cognitive processing of the Chinese and the Japanese languages*.

[CR22] Li H, Chen HC, Chen HC (1997). Processing of radicals in Chinese character recognition. *Cognitive processing of Chinese and related Asian languages*.

[CR23] Liu PD, Chung KKH, McBride-Chang C, Tong X (2010). Holistic versus analytic processing: Evidence for a different approach to processing of Chinese at the word and character levels in Chinese children. Journal of Experimental Child Psychology.

[CR24] Miles LK, Nind LK, Macrae CN (2010). Moving through time. Psychological Science.

[CR25] Ouellet M, Santiago J, Funes MJ, Lupiáñez J (2010). Thinking about the future moves attention to the right. Journal of Experimental Psychology: Human Perception and Performance.

[CR26] Ren C (2010). *Mirror reading of Chinese words: An ERP study* (doctorial dissertation).

[CR27] Santiago J, Román A, Ouellet M, Rodríguez N, Pérez-Azor P (2010). In hindsight, life flows from left to right. Psychological Research.

[CR28] Taft M, Zhu XP, Peng DL (1999). Positional specificity of radicals in Chinese character recognition. Journal of memory and Language.

[CR29] Tong X, Yip JHY (2015). Cracking the Chinese character: radical sensitivity in learners of Chinese as a foreign language and its relationship to Chinese word reading. Reading and Writing.

[CR30] Torralbo A, Santiago J, Lupiáñez J (2006). Flexible conceptual projection of time onto spatial frames of reference. Cognitive Science.

[CR31] Tsang Y-K, Chen H-C (2009). Do position-general radicals have a role to play in processing Chinese characters?. Language and Cognitive Processes.

[CR32] Tse CS, Altarriba J (2008). Evidence against linguistic relativity in Chinese and English: A case study of spatial and temporal metaphors. Journal of Cognition and Culture.

[CR33] Tversky B, Kugelmass S, Winter A (1991). Crosscultural and developmental-trends in graphic productions. Cognitive Psychology.

[CR34] Ulrich R, Eikmeier V, de la Vega I, Ruiz Fernandez S, Alex-Ruf S, Maienborn C (2012). With the past behind and the future ahead: Back-to-front representation of past and future sentences. Memory and Cognition.

[CR35] Vallesi A, Weisblatt Y, Semenza C, Shaki S (2014). Cultural modulations of space-time compatibility effects. Psychonomic Bulletin & Review.

[CR36] Walker EJ, Bergen BK, Núñez RE (2017). The spatial alignment of time: Differences in alignment of deictic and sequence time along the sagittal and lateral axes. Acta Psychologica.

[CR37] Weger UW, Pratt J (2008). Time flies like an arrow: Space-time compatibility effects suggest the use of a mental time-line. Psychonomic Bulletin & Review.

[CR38] Wu Y, Mo D, Tsang YK, Chen HC (2012). ERPs reveal sub-lexical processing in Chinese character recognition. Neuroscience Letters.

[CR39] Yang W, Sun Y (2016). English and Mandarin speakers’ mental representations of time: Some new evidence about the language-thought relationship. Review of Cognitive Linguistics.

[CR40] Yang W, Sun Y (2016). A monolingual mind can have two time lines: Exploring space-time mappings in Mandarin monolinguals. Psychonomic Bulletin & Review.

[CR41] Yin Y (2016). *The time course research of global and local processing in Chinese word recognition* (doctorial dissertation).

[CR42] Zhou L, Peng G, Zheng H-Y, Su I-F, Wang WS-Y (2013). Sub-lexical phonological and semantic processing of semantic radicals: a primed naming study. Reading and Writing.

[CR43] Zhou T, Xu Y, Yang Y (2008). A comparative study of mirror writing by normal participants and aphasic patients: A new perspective. Linguistic Sciences.

